# Primary non-Hodgkin lymphoma of liver

**DOI:** 10.3747/co.v16i4.443

**Published:** 2009-08

**Authors:** A. Masood, S. Kairouz, K.H. Hudhud, A.Z. Hegazi, A. Banu, N.C. Gupta

**Affiliations:** * Cancer Care Center of Frederick, Frederick, MD, U.S.A; † Deccan College of Medical Sciences, Hyderabad, India; ‡ Frederick Imaging Center, Frederick, MD, U.S.A

**Keywords:** Primary hepatic lymphoma, non-Hodgkin lymphoma, liver, rituximab

## Abstract

Primary non-Hodgkin lymphoma (nhl) of liver is a very rare malignancy. Here, we report the case of a 65-year-old man who presented with constipation and right groin pain of 2 months’ duration. A computed tomography (ct) scan of the abdomen incidentally detected multiple hypodense nodules in both lobes of the liver. Diagnosis of primary nhl of liver was made using ultrasound-guided biopsy. Extensive investigations—which included bone marrow biopsy; fluorescence *in situ* hybridization; flow cytometry; ct scan of chest, abdomen, and pelvis; and whole-body positron-emission tomography—showed no involvement of bone marrow, lymph nodes, spleen, or any other organ. The patient is currently being treated with a chop-r (cyclophosphamide–doxorubicin–vincristine–prednisolone/rituximab) regimen. The case has many unique features, including normal liver function tests, especially that for lactate dehydrogenase; no type B symptoms; and negative serology for viruses. The case demonstrates that primary hepatic lymphoma should be considered in the differential diagnosis of space-occupying liver lesions in presence of normal levels of alpha-fetoprotein and carcinoembryonic antigen. The literature is extensively reviewed.

## 1. INTRODUCTION

Primary hepatic lymphoma (phl) is a very rare malignancy 1. Although the liver contains lymphoid tissue, host factors may make the liver a poor environment for the development of malignant lymphoma 2. We present an interesting case of primary non-Hodgkin lymphoma (nhl) originating in liver. A literature review of clinical features, diagnosis, and management is also provided.

Written informed consent was obtained from the patient for publication of this case report.

## 2. CASE PRESENTATION

A 65-year-old man presented to his primary care physician in August 2008 with a 2-month history of constipation and occasional right groin pain. His history was significant for right inguinal hernia repaired in 1970. Patient denied any fever, night sweats, vomiting, chest pain, abdominal pain, diarrhea, blood in stools, or weight loss.

The patient’s history was also significant for hypothyroidism, hypertension, gout, type 2 diabetes mellitus, hyperlipidemia, and mitral valve disease without regurgitation. Current medications included lisinopril, insulin, fluoxetine, atorvastatin, thyroxin, metformin, and hydrochlorothiazide.

**FIGURE 1:**
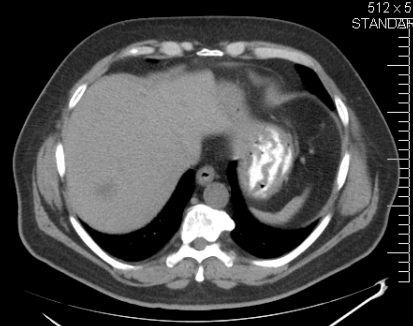
Computed tomography scan of abdomen, showing a hypodense nodule in the right lobe of the liver.

**FIGURE 2:**
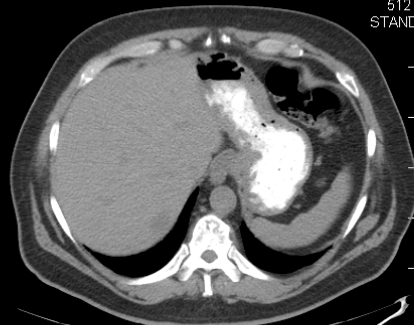
Computed tomography scan of abdomen, showing multiple hypodense nodules in the liver.

**FIGURE 3:**
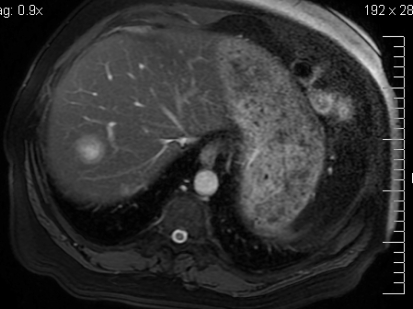
Magnetic resonance imaging of the abdomen, showing a mass in the right lobe of the liver. No lymphadenopathy or splenomegaly is seen.

A physical examination was unremarkable. The liver and spleen were normal in size. No superficial lymphadenopathy was present. Laboratory results included hemoglobin 13.3 g/dL and a white cell count of 5.5 × 10^9^/L, with a normal differential. Alanine aminotransferase, aspartate aminotransferase, alkaline phosphatase (alp), and lactate dehydrogenase (ldh) were within normal limits. Levels of serum alpha-fetoprotein and carcinoembryonic antigen (cea) were not elevated. Serology was negative for hiv and for the hepatitis C (hcv) and B (hbv) viruses. Serum calcium was within normal limits. In September 2008, a ct scan of abdomen and pelvis showed multiple hypodense nodules in the both lobes of the liver, without the classical enhancement pattern for hemangioma. The pancreas, spleen, and biliary tract were normal (Figures 1 and 2). Radiography and ct scan of chest did not reveal any mediastinal lymphadenopathy. Magnetic resonance imaging further confirmed the ct findings (Figure 3).

In October 2008, the patient was referred to our cancer care centre for further management. Imaging by positron-emission tomography was ordered and showed hypermetabolic activity (standardized uptake value up to 9.8) in a single lesion present in the right lobe of the liver.

**FIGURE 4:**
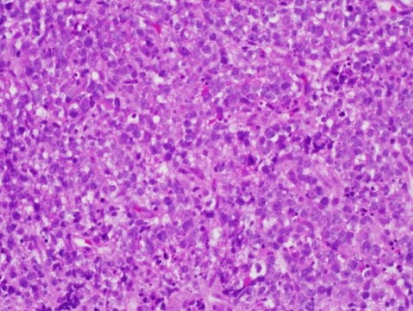
A photomicrograph shows diffuse infiltration by sheets of small-to-intermediate atypical cells. Some cells have evident nucleoli.

**FIGURE 5:**
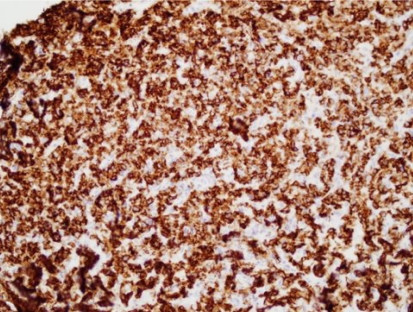
A photomicrograph shows tumour cells positive for CD20, proving B-cell lineage.

**FIGURE 6:**
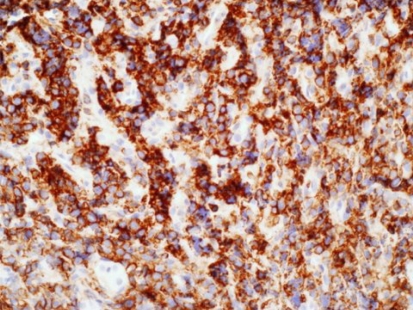
A photomicrograph shows tumour cells positive for CD45.

Histologic analysis after ultrasound-guided biopsy of the lesion showed diffuse infiltrates and small-to-intermediate atypical cells consistent with lymphoma (Figure 4). Immunostaining of the tumour cells showed reactivity for CD20, CD5, CD45, and Bcl6 (Figures 5 and 6).

Bone marrow biopsy showed normal cellularity with maturing tri-lineage hematopoiesis in normal proportion. No histologic or immunophenotypic evidence of B-cell lymphoma was present. Flow cytometry showed no phenotypic evidence of nhl. Fluorescent *in situ* hybridization demonstrated no evidence of t(11;14), trisomy 11, *BCL2* rearrangement, or *BCL6* breakpoint translocation. Flow cytometry and serum protein electrophoresis showed suppressed immunoglobulin M at 47 mg/dL, without monoclonal protein.

The patient was diagnosed with primary large B-cell lymphoma stage ie of liver, given that no additional foci of lymphoma were found anywhere else in the body. To date, the patient has received 4 cycles of cyclophosphamide–doxorubicin–vincristine–prednisone/rituximab (chop-r) and is planned for total of 8 cycles of chop-r.

## 3. DISCUSSION

Primary hepatic lymphoma is defined as lymphoma either confined to the liver or having major liver involvement 3. It represents less than 1% of all extranodal lymphomas 4.

The exact cause of phl is unknown, although viruses such as hbv, hcv, and Epstein–Barr have been implicated 3. There appears to be a strong association between primary hepatic nhl and hcv 5. Hepatitis C is found in 40%–60% of patients with phl 4. The possible roles of hcv, cirrhosis, and therapeutic interferon in lymphomagenesis remain hypothetical 6; however, our patient was not positive for hcv or hbv.

Primary hepatic lymphoma is twice as frequent in men as in women, and the usual age at presentation is 50 years 3. Presentations vary from the incidental discovery of hepatic abnormalities in otherwise asymptomatic patients (as in our case), to onset of fulminant hepatic failure with rapid progression of encephalopathy to coma and death 5. Symptoms are usually nonspecific, with most patients reporting right upper quadrant and epigastric pain, fatigue, weight loss, fever, anorexia, and nausea. Hepatomegaly is frequent, and jaundice is an occasional finding at physical examination 1.

Based on liver infiltration, phl can be subdivided into nodular or diffuse types 1. The pattern of liver infiltration has no prognostic value 4. Similarly, the disease may be of either T- or B-cell origin 1. Most phl corresponds to a larger cell type and demonstrates a B-cell immunophenotype 6. Other histologic subtypes of phl include high-grade tumours (lymphoblastic and Burkett lymphoma, 17%), follicular lymphoma (4%), diffuse histiocytic lymphoma (5%), lymphoma of the mucosa-associated lymphoid tissue type, anaplastic large-cell lymphoma, mantle cell lymphoma, and T-cell-rich B-cell lymphoma 7.

Patients with phl typically have abnormal liver function tests, with elevation of ldh and alp 4. Elevated ldh, with normal alpha-fetoprotein and cea, remains a valuable biologic feature 6; but in our case, all three markers were negative. For unknown reasons, hypercalcemia is present in about 40% of patients 4,7.

On ultrasound, phl lesions are hypo-echoic relative to normal liver. Imaging by ct shows hypo-attenuating lesions and rim enhancement after contrast. Findings on mri are variable 8; however, a few authors have described hypo-intense T1-weighted images and hyper-intense T2-weighted images 9.

Liver biopsy remains the most valuable tool for diagnosis of phl. If a discrete mass is not visible on imaging for percutaneous liver biopsy (plb), the transjugular approach may be reasonable. A recent review indicated that transjugular liver biopsy can be used to obtain adequate tissue samples and that major complications and mortality rates are similar to those with plb 3. For phl diagnosis, tumour must be confined to liver, without involvement of spleen, lymph nodes, bone marrow, or other lymphoid structures 7.

Most patients are treated with chemotherapy, with some physicians employing a multimodality approach that also incorporates surgery and radiotherapy 10. The standard treatment for patients with diffuse large B-cell lymphoma is chop. The addition of rituximab, a chimeric mouse–human monoclonal antibody targeting the pan-B-cell antigenic marker CD20 (the first monoclonal antibody licensed for use in the treatment of cancer 11), to the chop regimen, when given in 8 cycles, augments the complete response rate and prolongs event-free and overall survival in elderly patients with diffuse large B-cell lymphoma, without a clinically significant increase in toxicity 12. For patients with diffuse large B-cell nhl (including follicular nhl), several large-scale prospective randomized trials have established prolongation of remission when rituximab is incorporated into first-line treatment 13.

Poor prognostic features include advanced age, constitutional symptoms, bulky disease, unfavourable histologic subtype, elevated levels of ldh and β^2^-microglobulin, a high proliferation rate, cirrhosis, and comorbid conditions 10.

## 4. CONCLUSIONS

Primary hepatic lymphoma should be considered in the differential diagnosis in a patient with space-occupying liver lesions and normal levels of alpha-fetoprotein and cea. Diagnosis of this condition is important. If the clinical picture is suspicious for phl, a liver biopsy should be obtained, because the disease is treatable, and with new therapeutic drugs such as rituximab, overall survival has improved for these patients.
